# Integrating Patient Perspectives Into the Digital Health Technology Readiness Framework: Delphi Study

**DOI:** 10.2196/71600

**Published:** 2025-08-20

**Authors:** Elisenda de la Torre, Cristina Montane, Olga Rubio, Laura Sampietro-Colom, Araceli Camacho-Mahamud, Inmaculada Grau-Corral

**Affiliations:** 1 Patient Advocate, Barcelona, Spain; 2 Hospital Clínic de Barcelona, Villarroel 170, Barcelona, 08036, Spain; 3 Fundación iSYS, Barcelona, Spain; 4 Fundació de Recerca Clínic Barcelona-Institut d’Investigacions Biomèdiques August Pi i Sunyer, Barcelona, Spain

**Keywords:** patient engagement, patient participation, technology readiness levels, patient-centered design, Delphi method, health technology assessment, digital health innovation, technology acceptance model, unified theory of acceptance and use of technology, health care innovation, consensus building, patient advocacy, multidisciplinary collaboration, user-centered frameworks, health care technology adoption

## Abstract

**Background:**

Digital health technologies—including mobile applications, telemedicine platforms, artificial intelligence, and eHealth tools—are transforming health care delivery by enhancing access, personalization, and efficiency. However, traditional technology readiness levels (TRLs), while widely used to assess technological maturity, do not explicitly account for patient involvement—an essential factor in usability, acceptability, and real-world effectiveness.

**Objective:**

This study aimed to integrate patient perspectives into the TRLs framework, specifically tailoring it for applications in digital health innovations. By developing a patient-centered dimension using a Delphi methodology, this study provides actionable insights to enhance usability, acceptance, and real-world effectiveness of digital health technologies such as mHealth apps, telemedicine platforms, and eHealth solutions.

**Methods:**

A Delphi methodology was applied, involving 24 Spanish-speaking experts from diverse disciplines, including patient advocacy, clinical care, public health, ethics, and digital health engineering. Experts evaluated patient involvement statements across 10 TRL stages using a 6-point Likert scale. Consensus was defined a priori as ≥75% agreement and a mean score of ≥4.5. The Delphi process included 2 iterative rounds, allowing for refinement of the content despite initial consensus.

**Results:**

The Delphi process finally included 2 rounds, achieving a final 83.3% participation rate (20 of 24 experts). In round 1, all 10 TRL statements reached the predefined consensus threshold, with median scores ranging from 5.0 to 6.0 (83.3% to 100%) and mean scores from 4.70 (TRL2, 78.3%) to 5.25 (TRL5, 87.5%). While consensus was achieved, the presence of variability and qualitative feedback—particularly in early-stage TRLs such as TRL2 (Idea)—motivated a second round for refinement. In round 2, revised statements incorporating expert feedback were re-evaluated. Agreement increased across all TRLs, with mean scores ranging from 5.00 (TRL2, 83.3%) to 5.65 (TRL5, 94.2%). In total, 4 TRLs (TRL3, TRL4, TRL5, and TRL10) received a median of 6.0, indicating a unanimous strong agreement. Key refinements included more precise patient roles in usability testing, co-creation, clinical protocol design, and implementation monitoring. The framework also integrates patient-reported experience measures and patient-reported outcome measures in TRLs 5, 7, and 8.

**Conclusions:**

The PULSO-Tech-Clinic (Patient Participation, User/Usability, Literacy, System, and Observatory) Model is the first framework to systematically embed patient perspectives within the TRLs. Although consensus was achieved in the first round, a second round allowed for methodological rigor and optimization of clarity and inclusivity. This validated model enhances alignment with real-world patient needs and supports the design, evaluation, and adoption of patient-centered digital health technologies. Further research should evaluate its adaptability in diverse health care systems.

## Introduction

Digital health technologies, including mHealth applications, telemedicine platforms, and eHealth solutions, have rapidly transformed health care delivery, promising enhanced access, efficiency, and personalization of care. However, their success heavily depends on patient acceptance and usability—areas where traditional technology readiness levels (TRLs) frameworks often fall short. Recognizing the pivotal role of patient engagement in achieving meaningful and positive outcomes, involving patients in the development and evaluation of health care technologies ensures that these innovations align more closely with real-world needs and preferences.

Established theoretical frameworks, such as the unified theory of acceptance and use of technology (UTAUT) [1], underscore the importance of aligning health care technologies with patient needs and expectations throughout the development process. The UTAUT model identifies key factors influencing technology user acceptance, including performance expectancy, effort expectancy, social influence, and facilitating conditions. By integrating patient-centered insights based on these factors, technology solutions can more effectively address real-world usability and acceptance issues, thereby increasing the likelihood of successful implementation and improved health outcomes [2].

Patients also contribute essential insights for health technology assessments, which can lead to more informed coverage and reimbursement decisions [3,4]. Additionally, platforms that enable patients to contribute directly to innovations are advancing patient-driven solutions with the potential for significant commercial impact [5].

A key aspect of technology development is measuring its maturity, mostly done through TRLs. TRLs provide a structured framework to evaluate technological progress, from identifying an initial need and conceptualizing a project to achieving market readiness [6-8]. However, traditional TRLs do not fully capture the patient experience, an essential factor for successful health technology integration.

### Technology Readiness Level

NASA developed the TRL framework in the 1970s to assess the maturity of space technologies, attributed largely to Dr. Stan Sadin, with formal adoption in the 1980s [6]. The TRL scale, ranging from basic research to fully operational systems, was later adopted by US agencies such as the Department of Defense and the Department of Energy [6,7]. Since the early 2000s, European organizations have also adopted TRLs, with the European Space Agency and European Commission using them to advance technological innovations across sectors [9,10]. Over time, the framework has been adapted for use in various fields, including health care and digital health, serving as a valuable tool for project management and technology development worldwide [11].

In health care and digital health, TRLs have gained international acceptance, particularly for assessing the readiness of digital therapeutics and medical technologies. For instance, the European Union’s Horizon 2020 program uses TRLs to evaluate the clinical implementation readiness of digital health innovations [10]. In the United States, the Food and Drug Administration uses TRLs to structure the regulatory pathway for digital therapeutics from initial concept to market release [2]. In the United Kingdom, the National Institute for Health and Care Excellence incorporates TRLs in their assessment of digital health solutions to determine their effectiveness and readiness for integration into the health care system [3]. National programs in countries such as Japan and South Korea similarly integrate TRLs to promote the commercialization of innovative health technologies [12,13].

The European Union offers a set of generic TRL definitions [10], along with specific guidelines for different technology assessments (software and medical devices included) [14]. The Center for Integration of Medicine and Innovative Technology (CIMIT) in Boston proposes a comprehensive evaluation framework for health technology development. This framework extends beyond the traditional technological dimension to include 3 additional dimensions: clinical, regulatory, and economic feasibility [15]. Through collaboration with CIMIT, EIT Health developed the Healthcare Innovation Cycle framework, which provides a checklist across 10 maturity levels and 4 domains to monitor specific solution types, including MedTech, Digital Health, Biomarker Diagnostics, and Biotech [16], “a Framework for Innovation in Healthcare”, n.d.

### Patient Involvement in Health Technologies

Incorporating patient perspectives in the design and development of health care technologies has become increasingly crucial for effectively preventing, screening, diagnosing, treating, rehabilitating, and monitoring diseases. Several fundamental reasons underscore the importance of patient involvement in TRLs. Patients bring unique insights into their needs, desires, and preferences. Their involvement ensures that developed technologies are not only clinically effective and safe but also practical and beneficial for everyday use [17-19]. When patients contribute to the design and development of new technologies, they are more likely to feel comfortable using them and adhere to treatment protocols, thereby significantly improving their overall health outcomes [20]. Patient participation can highlight real-world problems that clinical or technical teams might otherwise overlook from a purely clinical or technical perspective. This collaboration can prioritize needs that are most important to patients, which may not be apparent through technical analysis alone [21,22]. Early-stage patient involvement in technological development can increase process transparency, fostering trust among patients, health care providers, and technology developers [23,24]. Including patients from diverse demographic and socioeconomic backgrounds helps ensure that new technologies are beneficial to a wide range of individuals, including those with special needs or socioeconomic disadvantages. Integrating patient participation into TRLs is not only desirable but essential for ensuring that new health technologies are effective, safe, accessible, usable, acceptable, and practical for all users [25,26].

A patient-centered approach can significantly enhance the success of technological developments in health care, ensuring that innovations align with users’ real needs. Beyond improving adoption and adherence. This approach also provides a competitive advantage and differentiation for organizations involved in their design and development [27,28].

The PULSO-Tech-Clinic model was designed to complement the traditional TRL framework by adding a structured, patient-centered perspective. PULSO stands for Patient Participation, User/Usability, Literacy, System, and Observatory—5 key pillars that support the inclusion of patient needs, values, and real-world usability in digital health innovation. This dimension has been largely underrepresented in traditional TRL-based approaches.

This study applies the Delphi technique [29] to gather and refine expert consensus on incorporating patient experiences and technology usability considerations into TRL assessments. Through this structured approach, the PULSO model offers actionable guidance to support user-centered design, particularly during early development and evaluation stages. The Delphi technique allows for the inclusion of diverse perspectives while reducing individual bias through participant anonymity. Its transparent and iterative process reinforces the rigor and credibility of the resulting framework.

Rather than simply updating existing frameworks, the consensus exercise introduces a new patient-centered dimension that guides the integration of patient perspectives across all stages of innovation.

### Objective

The objective of the PULSO-Tech-Clinic Model is to introduce a novel, independent dimension focused on patient inclusion, applicable to TRL models. This framework aims to provide a more holistic and comprehensive evaluation of health-oriented technologies, emphasizing patient-centered approaches.

Initially targeted in Spain, the model’s application is intended for broader Spanish-speaking regions, with an aim for global relevance. This study seeks to establish patient-centered TRLs as a standard consideration, enhancing health technology assessments and driving patient-oriented innovation forward.

## Methods

### Preparation for the Delphi Study

Preparation for the Delphi study followed a systematic approach, beginning with a comprehensive literature review to identify existing guidelines, best practices, and conceptual frameworks, with a particular emphasis on the pivotal role of patient insights in driving advancements in digital health innovations. A multidisciplinary team of 6 researchers—including 2 patient advocates (one with a technology background and one without), 2 clinicians (one focused on patient participation and one on technology assessment), and 2 engineers specializing in digital health—participated in 2 semi-structured focus groups to synthesize this information. Through these sessions, the team identified key elements and priorities and ultimately developed preliminary statements. The research team refined these drafted statements—capturing essential aspects of the study topic—through expert review before distribution to the Delphi panelists. This preliminary work aimed to establish a standardized knowledge base and promote a shared understanding among all participants.

### Study Design, Participants Recruitment, and Consensus Criteria

As recommended in the literature [24,26-29], the research team established the following parameters prior to initiating the Delphi process. A minimum participation rate of 70% was required for each Delphi round. Consensus is defined as a participant agreement level of 75%. Specifically, it was considered achieved when a mean score of 4.5 or higher was obtained on a 6-point Likert scale [24]. To maintain participant engagement, the process will be limited to a maximum of 3 rounds.

A 6-point Likert scale was used to assess agreement with each item, ranging from 1 (“strongly disagree”) to 6 (“strongly agree”). This even-numbered format was intentionally chosen to eliminate a neutral midpoint and encourage respondents to take a position—an approach recommended in Delphi studies to facilitate clearer consensus building [30,31]. A 6-point scale also provides sufficient granularity to capture variations in opinion without overburdening respondents, as may occur with longer scales [32]. This balance of sensitivity and usability made the 6-point format suitable for our expert panel.

Regarding the panel size, a figure of 15‐30 experts is commonly recommended in Delphi studies to ensure sufficient diversity and depth of perspectives without overwhelming the analysis, with studies by Hsu and Sandford [33] and Skulmoski et al [34] supporting this range as optimal for reliable consensus.

The Delphi panel was purposively selected by the multidisciplinary research team based on expertise in clinical care, digital health, patient advocacy, innovation, ethics, and health policy. Although participants were not randomly recruited, selection aimed to ensure balanced representation from key stakeholder groups relevant to the development of the PULSO model. This strategy helped mitigate potential selection bias by incorporating a diverse range of professional perspectives.

Panelists did not receive any incentives for participation. They were invited to participate via email, with up to 3 contact attempts; no additional follow-up invitations or reimbursement for their time were provided.

### Procedure

To conduct the study, the research team invited a selected panel of Spanish-speaking experts to participate in a Delphi survey by email. This multidisciplinary panel—comprising patient advocates, clinicians, technology specialists, public health experts, and ethics professionals—was chosen to capture diverse perspectives on patient inclusion within TRLs. Selection emphasized expertise in patient-centered approaches, health innovation, and technology evaluation.

The Delphi process began after expert panel members accepted the invitation to participate by distributing an electronic questionnaire. The Delphi exercise proceeded with participants receiving a list of 10 TRLs, each TRL accompanied by (1) standard TRL definitions based on reference texts, (2) a proposed statement for patient involvement, defined by the research team, (3) a mandatory Likert scale to rate the proposal, and (4) an optional text field for suggesting alternative wording (only in the first round).

Content validation was integrated into the iterative Delphi process, in line with established practices when working with expert panelists. Rather than conducting a separate pilot phase, initial TRL statements were developed through a targeted review of authoritative sources and refined via internal focus groups involving clinicians, engineers, and patient advocates. This preparatory work ensured conceptual alignment with the study’s objectives and initial clarity of the items.

In addition, round 1 included open comment fields for each item, allowing panelists to provide qualitative feedback and suggest rewording. This feedback informed the refinement of statements for round 2. As noted in Delphi methodology literature, such iterative internal validation is acceptable in studies involving highly specialized panels, as it enables potential ambiguity to be addressed within the Delphi rounds themselves [29].

In *round 1*, the initial form containing the 10 proposed TRL statements was distributed to the panel of experts, who rated each statement on a Likert scale from 1 (strongly disagree) to 6 (strongly agree). Responses were collected and analyzed to evaluate initial consensus levels. Experts also had the opportunity to suggest alternative wording during this round. Based on these results, the research team refined the statements and incorporated feedback from the Likert scale data to prepare for the next round.

In *round 2*, and, if necessary, round 3, the expert panel received summarized feedback from the previous round. Participants were asked to reconsider their ratings based on the group’s feedback. The revised ratings were then collected and analyzed to identify areas of agreement and divergence.

As mentioned before, the Delphi exercise concluded when a proposal reached 75% consensus or after 3 rounds, whichever occurred first. Items that achieved consensus prior to the conclusion of the exercise were selectively included in subsequent rounds based on the research team’s discretion. This strategic inclusion served to refine and optimize the overall outcome. The flexible approach allowed for iterative refinement, balancing the need for consensus with the imperative of producing a scientifically rigorous and practically applicable framework.

To collect panelists’ responses, a Google Form was used, enabling automatic data recording into a spreadsheet for analysis. Quantitative data from the Likert scale ratings were processed to calculate summary statistics and identify levels of consensus. Free-text responses were analyzed qualitatively using thematic analysis to identify recurring patterns, suggestions, and potential improvements proposed by the panelists. This combination of quantitative and qualitative methods ensured a comprehensive synthesis of feedback provided in each round.

### Statistical Analysis

Quantitative data were analyzed using mean scores, medians, and interquartile ranges to assess consensus levels across TRL items. Box-and-whisker plots were generated to visualize agreement distributions. Qualitative data from free-text responses were thematically analyzed using a systematic coding approach, where recurring themes and suggestions were categorized to inform iterative revisions. This dual-method analysis ensures both quantitative rigor and rich, context-sensitive insights.

At the end of the Delphi exercise, panelists were contacted via email to communicate the results and thank them for their participation. The feedback provided included quantitative data and statistics. All feedback was presented anonymously to ensure the confidentiality of individual opinions and to support an unbiased evaluation of the results.

The reporting of this Delphi study was guided by the Appraisal of Consensus Reporting Criteria in Delphi Studies (ACCORD) checklist [35] to guide the development of this paper, ensuring comprehensive reporting of methodological details, enhancing clarity, and promoting transparency in the design, execution, and presentation of study findings.

### Ethical Considerations

This study did not require approval from a Research Ethics Committee, as it involved a Delphi panel of professionals and patient advocates participating in their expert capacity. No clinical patients or vulnerable individuals were involved, and no personal health or identifying data were collected. All participants provided informed consent via email after receiving detailed information about the study’s objectives, procedures, and their rights, including the voluntary nature of participation and the option to withdraw at any time. Participants' privacy and confidentiality were maintained throughout the process, and no identifiable information is presented in the results. No compensation was offered for participation, in accordance with local ethical standards. According to Spanish regulations and institutional guidelines for studies involving expert consultation without the collection of personal data, formal ethical approval was not required.

## Results

### Overview

The Delphi study was initiated in spring 2024, beginning with a comprehensive literature review. The research team conducted 2 group sessions, which were supplemented by collaborative online proposal development. The insights gathered during these sessions formed the empirical foundation for the initial Delphi proposal documentation.

Invitations to participate in the Delphi exercise were sent starting in May 2024. The first round of the Delphi process occurred from June 5 to June 16, while the second round was conducted from June 19 to July 3.

Of the 24 experts who initially agreed to participate in the Delphi exercise, 20 completed the study, representing an 83.3% participation rate that exceeded the 70% threshold established by the methodology. All dropouts occurred during the first round, while the remaining participants actively engaged throughout both rounds.

The final panel consisted of 20 experts (final N=20): 6 clinicians (30%), 6 experts in patient experience or participation (30%), 2 patient advocates (10%), 2 professionals from innovation and industry (10%), 1 ethics expert, 1 global health consultant, and 2 academic researchers. This composition reflects a deliberately multidisciplinary and cross-sectoral perspective.

### First Round

**Figure 1.**
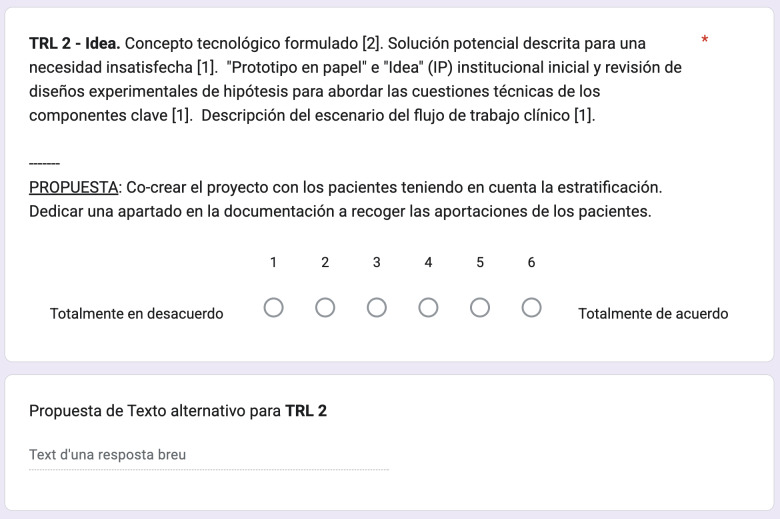
Example of a TRL content proposal.

The results of the first iteration are represented in Figure 1 using a box-and-whisker plot. This figure presents the evaluation scores for different texts representing TRL patient assessment proposed for the first round of the Delphi study, collected from the experts’ panel. The columns represented the TRLs from TRL1 (Need) to TRL10 (Standard of care), and the rows showed individual expert ratings on a Likert scale from 1 to 6.

**Figure 2.**
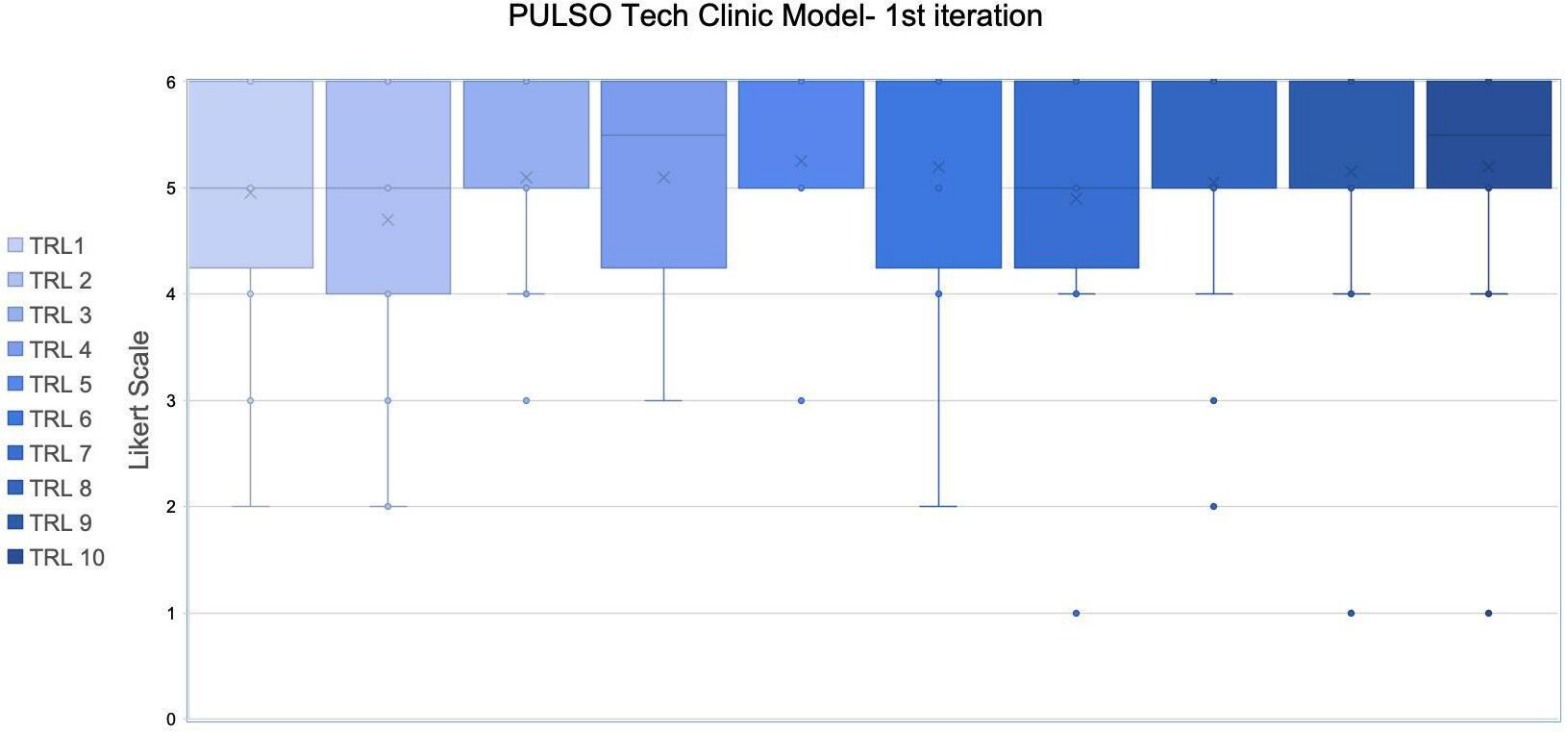
Box-and-whisker plot showing Likert scale ratings (1-6) for the PULSO Tech Clinic Model across 10 technology readiness levels (TRLs) during the first Delphi iteration. Boxes represent the interquartile range, horizontal lines indicate medians, and “X” marks the mean values. Whiskers show non-outlier ranges, with outliers plotted individually.

The median values for the patient’s sentence in each TRL were 5, except for TRL4 (SD 1.12) and TRL10 (SD 1.20), which had a median of 5.5, and TRL6, which had a median of 6. This indicated that, generally, experts rated most stages as satisfactory or better. The mean values for the TRLs ranged from 4.70 (SD 1.26, TRL2) to 5.25 (SD 0.91, TRL5), with TRL6 also being high at 5.20 (SD 1.11). This suggested a generally positive evaluation across all TRLs, with TRL2 having slightly lower average ratings. The range of values indicated some variability in expert opinions, especially noticeable in the early TRLs like TRL2 (Idea) and TRL3 (Proof of concept), where some lower ratings (eg, 2 and 3) were present (Figure 2).

Although there was a high consensus in the first round, there were also several outliers and concordant proposals to improve the text, which were incorporated into the proposal, and we proceeded to the second round.

### Second Round

In the second round, the proposal’s text was reviewed with a focus on clarity and precision, particularly for the TRLs with lower consensus. Based on expert feedback, textual and structural improvements were made to enhance the clarity and inclusiveness of the TRL descriptions. The descriptions were further refined to ensure comprehensiveness and inclusivity, with adjustments made to better communicate the roles and responsibilities of different stakeholders involved in the process. By emphasizing stakeholder engagement, continuous feedback, and monitoring, the proposal was refined to align with best practices and remain relevant to the community.

**Figure 3.**
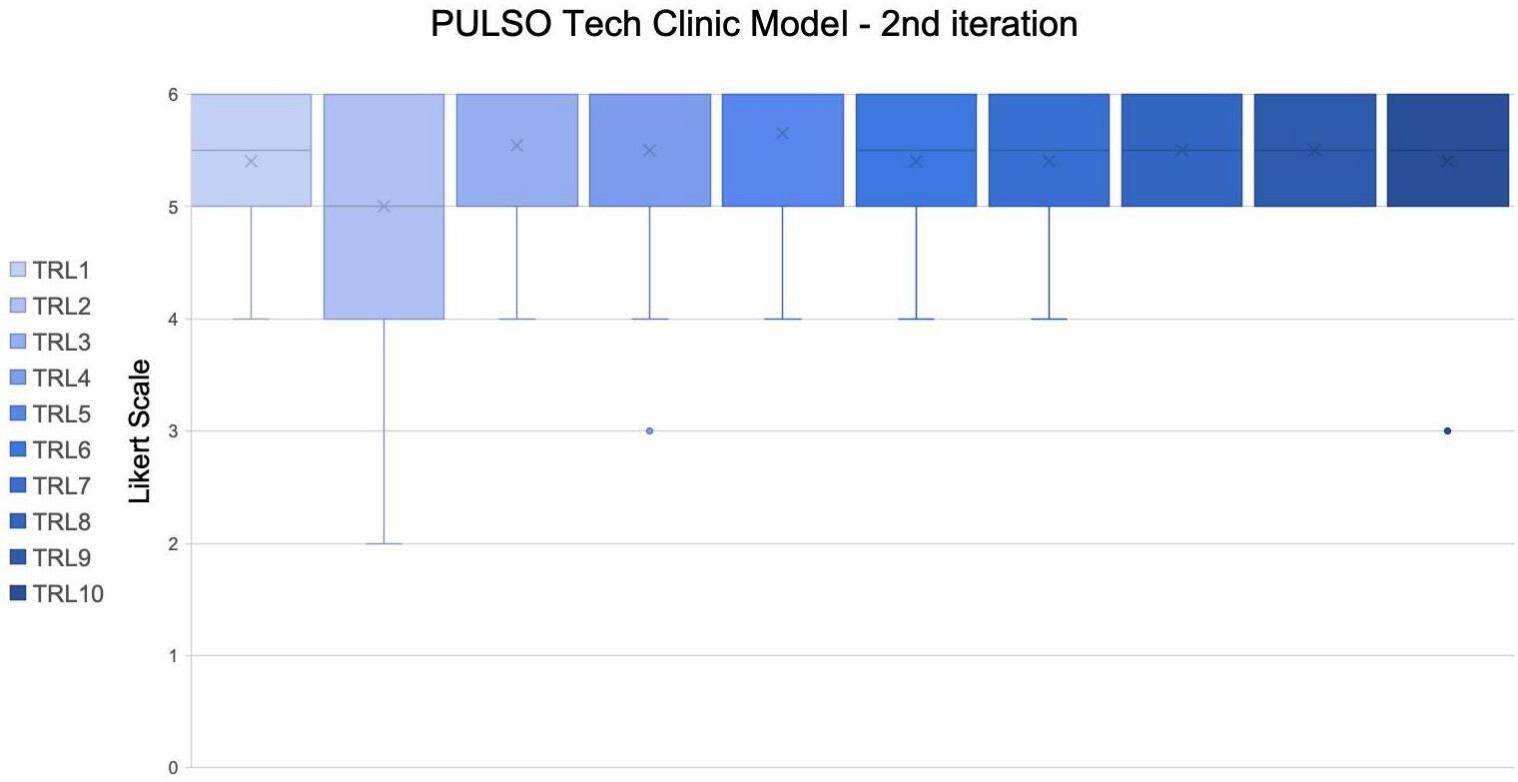
Box-and-whisker plot showing Likert scale ratings (1-6) for the PULSO Tech Clinic Model across 10 technology readiness levels (TRLs) during the second Delphi iteration. Boxes represent the interquartile range, horizontal lines indicate medians, and “X” marks the mean values. Whiskers display non-outlier ranges, with reduced variability compared to the first iteration, indicating improved consensus across all TRLs.

The initial proposal was adapted based on the panel of experts’ suggestions, where no discrepancies were identified. The research group proceeded with the inclusion of detailed steps and specific actions required at each TRL stage in the new questionnaire for round 2. In this round, open proposals were not available, and the only option was to provide appraisal using the Likert scale. The result of the second round is shown in Figure 3.

The last 2 rows summarized the median and mean values for each TRL: The median values for all TRLs were consistently high, with TRL3, TRL4, TRL5, and TRL10 having a median of 6, and the rest of the TRLs having a median of 5.5 or 5. This indicated that the experts generally rated the stages as satisfactory or better. The mean values for the TRLs ranged from 5.00 (TRL2) to 5.65 (TRL5), with TRL3 having a mean of 5.55. This suggested a generally positive evaluation across all TRLs, with no TRL falling below a mean value of 5.00.

### Final Proposal

The results of the consensus exercise are illustrated in Table 1, which shows the agreed-upon content regarding patient involvement at each TRL. This table reflects the consensus reached by the expert panel during the final round of the Delphi exercise.

**Table 1.** Result of Delphi exercise.

TRL	Original (Spanish)	Translation (English)
1. Need	Identificación y estratificación inclusiva del paciente y/o cuidador, teniendo en cuenta aspectos tales como la fase o grado de la enfermedad, el sexo, la edad, los recursos socioeconómicos, etc.	Inclusive identification and stratification of the patient and caregiver, taking into account aspects such as the stage or degree of the disease, gender, age, socioeconomic resources, etc.
2. Idea	Co-crear el proyecto con los pacientes teniendo en cuenta la estratificación. Destacar la colaboración de pacientes y/o familiares en la documentación del proyecto.	Co-create the project with patients taking into account stratification. Emphasize the involvement of patients and family members in the documentation of the project.
3. Proof of concept (PoC)	Diseñar y validar la “prueba de concepto” con el grupo de pacientes y/o cuidadores objetivo involucrándolos activamente en el proceso. Registro sistemático de sus aportaciones	Design and validate the “proof of concept” with the target group of patients and caregivers by actively involving them in the process. Systematically record their input
4. Proof of feasibility (PoF)	Realizar pruebas objetivas de usabilidad, funcionalidad y utilidad con el grupo de pacientes y/o cuidadores objetivo. Identificar posibles mejoras y limitaciones.	Conduct objective usability, functionality, and utility tests with the target group of patients and caregivers. Identify potential improvements and limitations.
5. Proof of value (PoV)	Preparar el proceso de evaluación con los pacientes y/o cuidadores. Probar la solución mejorada con un grupo representativo de ellos. Crear conjuntamente las instrucciones de uso, revisar la claridad de textos legales y consentimientos y confección, si procede, de cuestionarios PREMs y PROMs^a^.	Prepare the evaluation process with patients and caregivers. Test the improved solution with a representative group of them. Work together to create the instructions for use, check the clarity of legal texts and consents, and prepare PREMs and PROMs questionnaires, if necessary.
6. Initial clinical trials (ICT)	Incluir a los pacientes y/o cuidadores en el diseño del protocolo del ensayo clínico. Invitar a participar a los pacientes objetivo en los ensayos clínicos iniciales.	Involve patients and caregivers in the design of the clinical trial protocol. Invite target patients to participate in early clinical trials
7. Validation of solution (VoS)	Incluir a los pacientes y/o cuidadores en el diseño del estudio, en las pruebas de eficacia clínica y de estrés del desarrollo tecnológico, y en la medición de la usabilidad, la adherencia y los PREMs y PROMs, para propiciar que los resultados sean relevantes y significativos para la comunidad que se va a beneficiar.	Involve patients and/or caregivers in study design, clinical effectiveness, and stress testing of technology development, and measurement of usability, adherence, and PREMs and PROMs to ensure that results are relevant and meaningful to the community that will benefit.
8. Approval and launch (A&L)	Revisión conjunta con asociaciones y defensores de pacientes, incluyendo un mecanismo de seguimiento para evaluar la adopción y adherencia. Valoración de resultados de eficiencia clínica, así como de experiencia del paciente y calidad de vida. Seguimiento continuo para la detección de problemas, permitiendo mejoras continuas.	Joint review with patient associations and advocates, including a monitoring mechanism to assess adoption and adherence. Assessment of clinical efficiency outcomes, as well as patient experience and quality of life. Ongoing monitoring to detect problems allowing for continuous improvement.
9. Clinical use (Use)	Difusión entre las asociaciones de pacientes y/o cuidadores. Implementar herramientas de feedback que permitan identificar oportunidades de mejora, así como identificar barreras de aquellos que no la utilizan, para así adaptar y mejorar la solución.	Dissemination among patient and caregiver associations. Implement feedback tools that allow for identifying opportunities for improvement, as well as identifying barriers for those who do not use it, in order to adapt and improve the solution.
10. Standard of care (SoC)	Recomendación por parte de organismos públicos y en jornadas de pacientes.	Recommendation by public bodies and at patient conferences.

a PREMs: patient-reported experience measurements; PROMs: patient-reported outcomes measurements. The table presents the results in the original Spanish alongside their English translations for clarity and transparency.

Table 1 summarizes the results of the Delphi study, outlining patient involvement at each TRL. The table presents the original Spanish text alongside its English translation, covering all TRLs from TRL1 (Need) to TRL10 (Standard of care).

The PULSO framework incorporates patient perspectives across all TRL stages, emphasizing actionable roles in digital health innovation: TRL 1-3 (early stages): co-creation with patients to identify unmet needs and design features aligned with user preferences; and TRL 7‐9 (late stages): validation in real-world settings, addressing cultural adaptability and monitoring adoption barriers.

## Discussion

### Principal Findings

The results of our study demonstrate a strong expert consensus regarding the importance of patient involvement across all TRLs in the development and evaluation of health care technologies. This is evidenced by the high median and mean values achieved across all TRLs, emphasizing the effectiveness of involving patients and other stakeholders throughout the process. Many studies recommend involving patients from the beginning of the development process and highlight the need to incorporate the patient’s voice into the design of digital health technologies [36-40].

Reflecting on how this can improve the design and development of technologies, we can consider each TRL stage. For instance, at the initial stages (TRL 1‐3), patient input can guide the identification of unmet needs and potential usability issues. In the mid-stages (TRL 4‐6), patients can help refine prototypes and ensure that the technology addresses real-world challenges. At the later stages (TRL 7‐9), patient feedback can be crucial for validating the technology in real-world settings and ensuring its acceptance and adoption. This study has the strengths of having co-created with patients the definition of each TRL item, and it may be for this reason that the degree of agreement is greater in Delphi groups.

The Delphi exercise results align with the UTAUT [1], emphasizing the integration of patient needs and expectations into health care technology development. The stepwise recommendations across the TRLs provide a structured approach to addressing user acceptance factors, such as performance expectancy and facilitating conditions. Early TRLs focus on inclusive identification and co-creation with patients, ensuring relevance and usability, while middle TRLs emphasize patient involvement in usability testing, feedback collection, and tool creation, such as patient-reported experience measures and patient-reported outcome measures. Later TRLs incorporate mechanisms for monitoring adoption and adherence, fostering continuous improvement. This framework highlights the critical role of patient inclusion at every stage, operationalizing UTAUT principles and enhancing the usability and acceptance of health care innovations.

Patients are defined as one of the crucial stakeholders of health care and decision-making, and this shows the need to involve them in the treatment process [41]. Over the past 2 decades, the use of patient experiences in assessing the quality of care from a patient perspective has received more attention, which shows that it is not only possible to involve patients in the delivery or redesign of health care, but such engagement can lead to a reduction in the number of hospitalizations, improved effectiveness, efficiency, quality of health services, quality of life, and responsiveness [42].

Unlike existing innovation frameworks such as CIMIT or the Healthcare Innovation Cycle—which primarily emphasize clinical validation, regulatory readiness, or implementation stages—the PULSO model introduces a structured layer of patient involvement across all TRL stages, including upstream phases like ideation, co-design, and early usability testing. This makes PULSO uniquely positioned to bridge the gap between technological development and real-world acceptability, ensuring that patient-centered criteria are integrated from the earliest stages of innovation.

However, there are challenges in involving patients at different TRL stages. From the patient’s perspective, there may be barriers such as a lack of understanding of technology, time constraints, or health issues. From the developers’ perspective, integrating patient feedback can be resource-intensive and may require specialized units, like the patient engagement unit at the hospital, which not all institutions have.

Referring to other works that support the integration of patients’ perspectives in medical research and technological development, Babac et al [43] highlight the importance of shared decision-making in health care, which aligns with our study’s emphasis on patient participation. Similarly, van der Scheer et al [44].

Incorporating a patient’s perspective may help health care professionals to collaborate with patients to work together to improve health outcomes [45].

### Strengths and Limitations

#### Strengths

This study achieved a high participation rate among a panel of experts with relevant knowledge and interest in patient-centered digital health innovation, which strengthens the credibility and relevance of our findings. The use of the Delphi methodology allowed for a rigorous and structured consensus-building process, ensuring diverse and impartial input.

#### Limitations

The study’s limitations include the relatively small sample size, which was based on a convenience sample of experts. While this approach prioritized participants with specialized expertise, it may limit the generalizability of the findings. Additionally, most participants were from a single country, which could introduce potential biases, as health care systems and the inclination to include patients in development processes vary internationally.

### Future Directions

Further research is needed to explore the extent to which incorporating a patient perspective into TRL frameworks can impact the quality and effectiveness of health care services. Expanding the framework’s application across diverse health care settings and cultures would also enhance its adaptability and utility.

### Conclusions

To the best of our knowledge, this is the first study to incorporate the patient’s perspective within the TRLs. The results are both compelling and robust, highlighting the significant impact of patient involvement in the development and evaluation of health care technologies.

Incorporating a patient dimension into the TRL scale can significantly enhance health care technology development and adoption by focusing on patient needs, preferences, and experiences. This integration fosters a patient-centered approach, improves usability and safety, increases acceptance, and facilitates personalized medicine. Ultimately, this can lead to better alignment with health care goals, improved health outcomes, and higher patient satisfaction while supporting regulatory approval and reimbursement processes.

## Abbreviations

ACCORD: Appraisal of Consensus Reporting Criteria in Delphi Studies
CIMIT: Center for Integration of Medicine and Innovative Technology
PULSO: Patient Participation, User/Usability, Literacy, System, and Observatory
TRL: technology readiness level
UTAUT: unified theory of acceptance and use of technology
